# Accuracy of the painDETECT screening questionnaire for detection of neuropathic components in hospital-based patients with orofacial pain: a prospective cohort study

**DOI:** 10.1186/s10194-018-0932-5

**Published:** 2018-11-06

**Authors:** Daniyal J Jafree, Joanna M Zakrzewska, Saumya Bhatia, Carolina Venda Nova

**Affiliations:** 1 Faculty of Medical Sciences, University College London, London, UK; 2 Eastman Dental Institute, UCLH NHS Foundation Trust, London, UK

**Keywords:** Screening tool, Orofacial pain, Trigeminal neuralgia, Temporomandibular disorder, Neuropathic pain, Questionnaire, Diagnosis

## Abstract

**Background:**

Better tools are required for the earlier identification and management of orofacial pain with different aetiologies. The painDETECT questionnaire is a patient-completed screening tool with utility for identification of neuropathic pain in a range of contexts. 254 patients, referred from primary care for management of orofacial pain and attending a secondary care centre, were prospectively recruited, and completed the painDETECT prior to consultation. The aim of this study was to determine the accuracy of the painDETECT to detect neuropathic components of orofacial pain, when compared to a reference standard of clinical diagnosis by experienced physicians, in a cohort of hospital-based patients.

**Results:**

For the 251 patients included in the analysis, the painDETECT had a modest ability to detect neuropathic components of orofacial pain (AUROC, 0.63; 95% CI, 0.58–0.70; *p* = 0.001). Patients with orofacial pain diagnoses associated with neuropathic components had higher painDETECT scores than those with non-neuropathic components. However, the painDETECT was weaker at distinguishing patients with mixed pain types, and multiple diagnoses were associated with poor accuracy of the painDETECT.

**Conclusion:**

In secondary care settings, the painDETECT performed modestly at identifying neuropathic components, and underestimates the complexity of orofacial pain in its mixed presentations and with multiple diagnoses. Prior to clinical applications or research use, the painDETECT and other generic screening tools must be adapted and revalidated for orofacial pain patients, and separately in primary care, where orofacial pain is considerably less common.

**Electronic supplementary material:**

The online version of this article (10.1186/s10194-018-0932-5) contains supplementary material, which is available to authorized users.

## Introduction

Accurate diagnosis of orofacial pain (OFP) is essential for appropriate patient management in primary and secondary care. Acquisition of a detailed pain history and examination directs diagnoses and treatment [1]. However, diagnosis of OFP is complex. Certain types of OFP are musculoskeletal in origin, such as temporomandibular disorders (TMD), others are neuropathic, such as trigeminal neuralgia (TN) and nerve injury-post dental extraction, whereas some have an unknown aetiology, such as chronic (persistent) idiopathic facial pain (CIFP). Mixed pain syndromes may also exist, where, rather than a binary distinction, pain may exist on a continuum of ‘more or less neuropathic’ [2, 3]. Due to a limited understanding of the pathophysiology of these processes, and the possibility of multiple OFP diagnoses occurring within the same patient, misdiagnosis and inappropriate referral of these patients is common, particularly for non-specialist clinicians [4, 5]. The management of musculoskeletal compared to neuropathic origin varies. For example, though commonly prescribed in primary and secondary care, non-steroidal anti-inflammatory medications are not recommended for neuropathic pain [6]. Moreover, the management of neuropathic pain is challenging, as patients are frequently unresponsive to drug treatment [7]. Earlier recognition and distinction of the aetiology of OFP in patients is needed, particularly due to the substantial patient burden and interference with daily living that some diagnoses may have [8].

Patient-completed screening questionnaires may supplement the recognition and clinical diagnosis of OFP in a variety of settings. These are paper-based or electronic tools that are easily administered to patients. In differentiating between common dental conditions and unknown OFP diagnoses [9], screening questionnaires may be useful for the earlier triaging of OFP patients to appropriate secondary or tertiary care pathways. However, it is important that these tools are validated for use in different settings, including primary or secondary care and epidemiological surveys. Such screening questionnaires may also be available to patients to complete and score over the internet, with no input from health care professionals, which adds to the importance of determining if they can accurately recognise different OFP diagnoses.

One such tool developed in 2006, the painDETECT screening questionnaire (PD-Q), uses a scoring method between − 1 and 38 to estimate the likelihood of a neuropathic pain component in patients. The PD-Q was originally designed to identify neuropathic components in back pain [10]. Since its conception, the PD-Q has been validated and translated into multiple languages, it is easy for patients to use, and has been shown to identify neuropathic pain components in different contexts, including lower back pain, arthritis, fibromyalgia, thoracotomy and malignancy [11]. Compared to other screening tools for neuropathic pain, the PD-Q does not require clinical examination, inquires about pain evoked by mild pressure and heat or cold [12] and thus has the potential to be used as a rapid pre-consultation tool to differentiate between aetiologies of OFP. To date, the PD-Q has been tested in populations of patients with specific OFP diagnoses. Elias and colleagues found that 34% of patients with post-traumatic trigeminal nerve injury at their centre obtained a PD-Q score of at least 19 [13]. More recently, Heo and colleagues applied the PD-Q to patients with burning mouth syndrome (BMS), and found a low sensitivity and high specificity for the identification of neuropathic pain components in this population [14]. Testing the PD-Q across a broad range of facial pain diagnoses is required to determine whether this tool would have utility as a screening tool for neuropathic pain in OFP. Our centre receives a heterogeneous group of patients with OFP [5], providing an opportunity to assess the PD-Q in a secondary care setting. The aim of this study was to determine the utility of the PD-Q to detect neuropathic pain in a hospital-based cohort of patients with OFP.

## Methods

### Design and setting

Given its diagnostic nature, this prospective, single-centre cohort study was conducted in concordance with the latest version of the STARD checklist for reporting studies of diagnostic accuracy [15]. Ethical approval was gained for the study from the South East London REC 3 Proportionate Review Sub Committee (Reference: 10/H0808/84). Patients were recruited at a London academic facial pain centre, which sees over 700 new patients a year, referred by primary care practitioners or specialists and in the oral surgery unit [5]. Prior to their appointment at our centre, patients routinely complete a series of questionnaires [16].

### Participants

Recruitment was conducted by three specialty dentists between 2010 and 2015, each completing their postgraduate studies. During the project phase of the dentists’ postgraduate studies, all patients referred from primary care, and attending OFP clinics and one oral surgery clinic, were consecutively recruited. Participants were excluded from the study if they: were below 18 years of age at consultation, had declined participation, were unable to complete the questionnaire without assistance. Participants with acute pain and those with more than one OFP diagnosis were recruited. From each participant, the following characteristics were planned, prior to PD-Q completion or consultation, and collected for each participant: age in years, gender, any secondary clinical diagnoses and the presence of anxiety or depression based on Hospital Anxiety and Depression Scale scores [17].

### Test methods

Participants completed a paper-based copy of the PD-Q prior to their consultation with the clinician. The questionnaires were collected by the specialty dentists, and not shown to the assessing clinicians. Uncompleted questionnaires were returned to the patient before consultation to encourage completion, but questionnaires remaining incomplete were excluded from analyses. The clinical diagnosis of each patient, serving as the reference standard of diagnosis, was obtained after a full assessment by an expert in pain medicine by means of a consultation, with a detailed pain history and clinical examination. Secondary diagnoses, classified as either orofacial pain or an alternative pre-existing non-orofacial diagnosis, were assigned to patient if necessary, but the primary diagnosis was classified as the predominant pain experienced. An independent clinician reviewed the initial diagnoses and confirmed these after initiation of a management plan. Clinical diagnosis was selected as a reference test, as it is presently the gold standard for diagnosis; based on the requirement of a detailed pain history and examination for differential diagnosis of OFP [18]. Clinical diagnoses were then grouped according to The International Classification of Headache Disorders [19], with a separate and specific classification applied for TMD [20]. Prior to analysis, clinical diagnoses were grouped into predominantly neuropathic, pain of a mixed aetiology with both neuropathic and non-neuropathic components, or non-neuropathic. At this stage, participants with a diagnosis not confirmed by an independent clinician were excluded from analysis.

The completed questionnaires were scored according to the methodology described in the original reports of the PD-Q [10]. Cut-offs were applied for analysis of the PD-Q as previously described. A PD-Q score ≤ 12 indicates a neuropathic component is not likely, whereas a score ≥ 19 indicates that a neuropathic component is likely. Between PD-Q scores of 12 and 19, neuropathic pain can be present, but is uncertain. Cut-offs were not applicable for the reference standard of clinical diagnosis. As the questionnaires were completed by each participant prior to consultation, clinicians were blinded to the results of the index test. Independent study investigators received clinical information, the results of the index test and reference standard.

### Analysis and statistics

As a previous study found the PD-Q to have an AUC of approximately 0.8 to distinguish BMS from nociceptive pain [14], it was anticipated that the PD-Q would have an accuracy of 80%, and it is required to estimate this figure to within 5% of the true population value. With a 95% confidence interval (CI), it was calculated that 246 patients were required for the study. The primary outcome of the study was the accuracy of the PD-Q for recognition of neuropathic pain components, compared with clinical diagnosis made by senior staff. This was determined using: sensitivity, specificity, predictive values, and receiver operating characteristics (ROC). For ROC analysis, the ‘test’ state was defined as patients with neuropathic pain or pain of mixed aetiology, whereas patients with non-neuropathic pain served as the ‘control’ state. ROC curves were drawn and the area under the curve (AUC) was calculated. The accuracy of the PD-Q was further analysed by comparing the PD-Q scores for patients with neuropathic, non-neuropathic or mixed pain using a Kruskal-Wallis test. Where a significant difference in PD-Q was observed across diagnoses, pairwise multiple comparisons with Bonferroni correction were used to calculate adjusted *p* values between individual diagnoses. The secondary outcome of the study related to factors influencing correct diagnosis of the PD-Q. This included determining Pearson’s correlation co-efficient (*r*) between PD-Q scores and each patient characteristic, and a stepwise multivariate logistic regression to independently determine the adjusted effect (using normalised β values) of each patient characteristic and PD-Q scores.

All continuous variables, where parametric, are presented as means with standard deviations (SD), and where non-parametric, are presented as medians with interquartile range (IQR). Categorical variables are presented numerically and as a percentage of the sample. *p* values less than or equal to 0.05 were considered statistically significant. 95% CIs were applied to all continuous outcomes, and percentages were calculated for categorical outcomes. All data were managed, analysed and graphed using IBM SPSS Statistics for Macintosh, Version 25.0 (IBM Corp., Armond, NY) and Prism for Macintosh, Version 7 (GraphPad Software Inc., San Diego, CA).

## Results

### Participants and characteristics

**Fig. 1:**
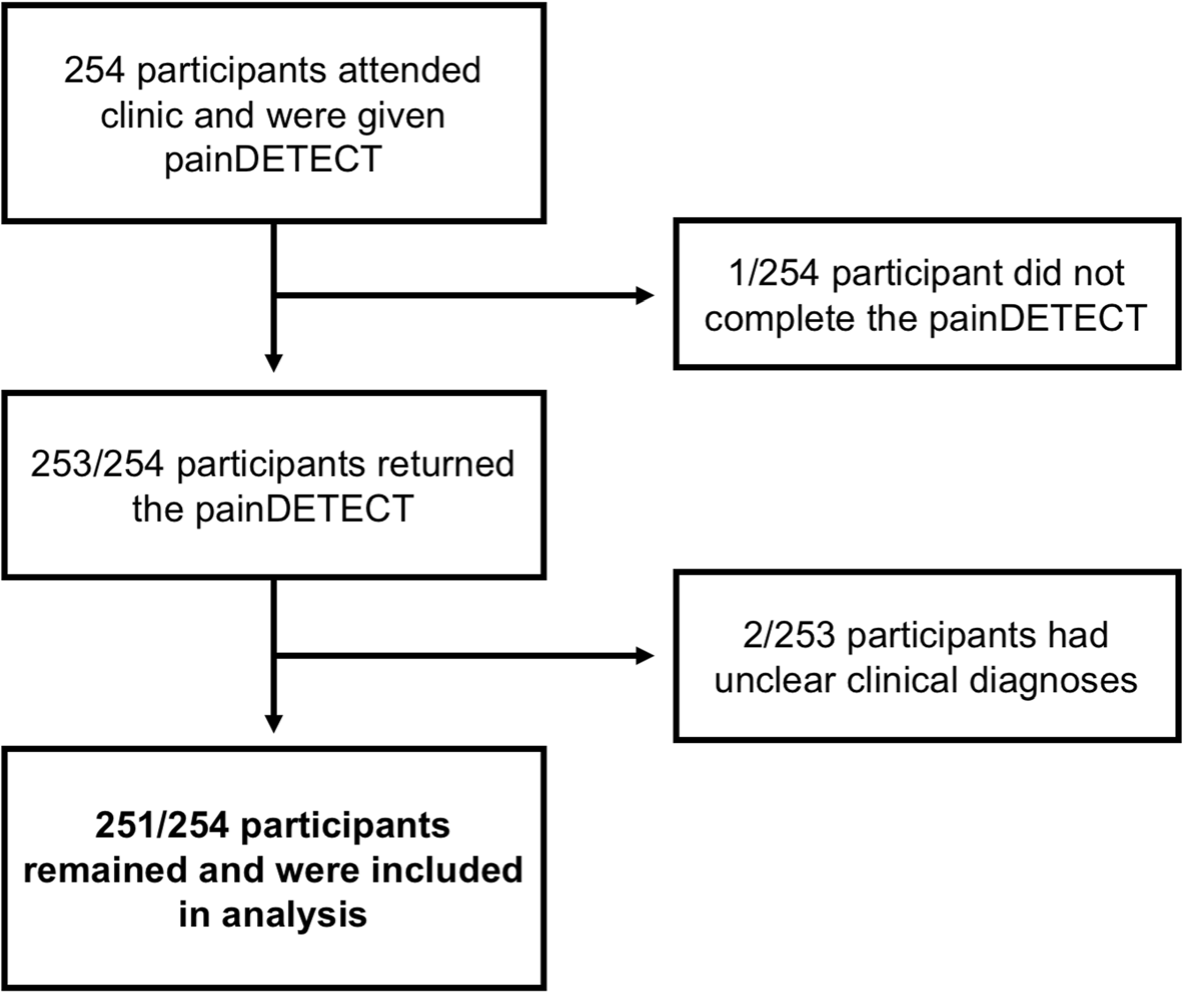
Participant flow diagram. One patient who did not complete the questionnaire had difficulty reading the questionnaire. The two patients with unclear clinical diagnoses were categorised as having orofacial pain of mixed aetiology

254 participants attended the facial pain clinic during recruitment periods between 2010 and 2015, and were given the PD-Q to complete prior to their appointment with the clinician (Fig. 1). All participants were subsequently seen by the facial pain team, who took the history and performed the examination to ascertain the clinical diagnosis. From the 254 patients, one patient was excluded due to non-completion of the questionnaire. From the remaining 253 patients, a further two were excluded due to discrepancy in the clinical diagnosis. Therefore, 251 out of 254 (98.8%) of patients were included in the analysis. Patient characteristics are presented in Table 1, stratified by the aetiology of OFP. The overall characteristics of the cohort of 251 participants were as follows: mean age, 47.3 (SD, 15.7); proportion of females, 191/251 (76.1%); proportion with a secondary diagnosis, 74/251 (29.4%, see Additional file 1: Table S1) and proportion with anxiety or depression, 48/250 (19.2%). The numbers of patients for each clinical diagnosis are shown in Table 2.

**Table 1:** Patients characteristics grouped by pain type

Patient characteristics	Neuropathic (*n* = 72)	Non-neuropathic (*n* = 137)	Mixed (*n* = 42)
Mean age in years (± SD)	54.0 ± 13.1	42.6 ± 15.6	51.1 ± 15.0
Female (%)	51 (70.8)	107 (78.1)	33 (78.6)
Secondary clinical diagnosis (%)	23 (31.9)	34 (24.8)	17 (40.5)
Anxiety or depression^a^ (%)	12 (16.7)	29 (21.2)	7 (16.7)
*Anxiety only*	*5*	*12*	*6*
*Depression only*	*5*	*10*	*0*
*Both anxiety and depression*	*2*	*7*	*1*

^a^: Anxiety or depression as determined by HADS scores

**Table 2:** Classification and frequency of OFP diagnoses

Neuropathic (*n)*	Non-neuropathic (*n)*	Mixed pain (*n)*
Trigeminal neuralgia (27)	Temporomandibular disorder (88)	Atypical odontalgia (20)
Trigeminal neuropathic pain (22)	Pericoronitis (8)^a^	Chronic idiopathic facial pain (13)
Burning mouth syndrome (6)	Psychosomatic pain (8)	Chronic post-dental treatment (6)
Trigeminal neuralgia with concomitant pain (5)	Migraine (5)	Post-radiotherapy (1)
Short unilateral neuralgiform headache attacks with autonomic features (5)	Pulpitis (4)^a^	Post-stroke (1)
Short-lasting unilateral neuralgiform headache with conjunctival injection and tearing (1) Post-herpetic neuralgia (2)	Acute post-dental treatment (3)^a^	Post-brain surgery (1)
Hemicrania continua (2)	Unspecified muscular (3)	
	Periodontitis (3)^a^	
Neuropathic post-trauma (1)	Tension headache (2)	
Facial pain with multiple sclerosis (1)	Hypervigilance (2)	
Tumour-associated neuropathic (1)	Temporal arteritis (1)	
	Non-odontogenic, persistent orofacial muscle pain (1)	
	Denture granuloma (1)^a^	
	Rheumatoid arthritis (1)	
	Parotitis (1)^a^	
	Dental abscess (1)^a^	
	Erythema migrans (1)	
	Post apicectomy (1)^a^	
	Insertion of dental implant (1)^a^	
	Fibromyalgia (1)	

^a^*:* Pain is dental in origin

#### Accuracy of the PD-Q for recognition of neuropathic pain components in orofacial pain

The PD-Q scores were calculated for each of the 251 participants, stratified by neuropathic, non-neuropathic or mixed aetiology. There was minimal time between administration of the PD-Q and subsequent appointment with a clinician. No participants experienced adverse events during the study period.

**Fig. 2:**
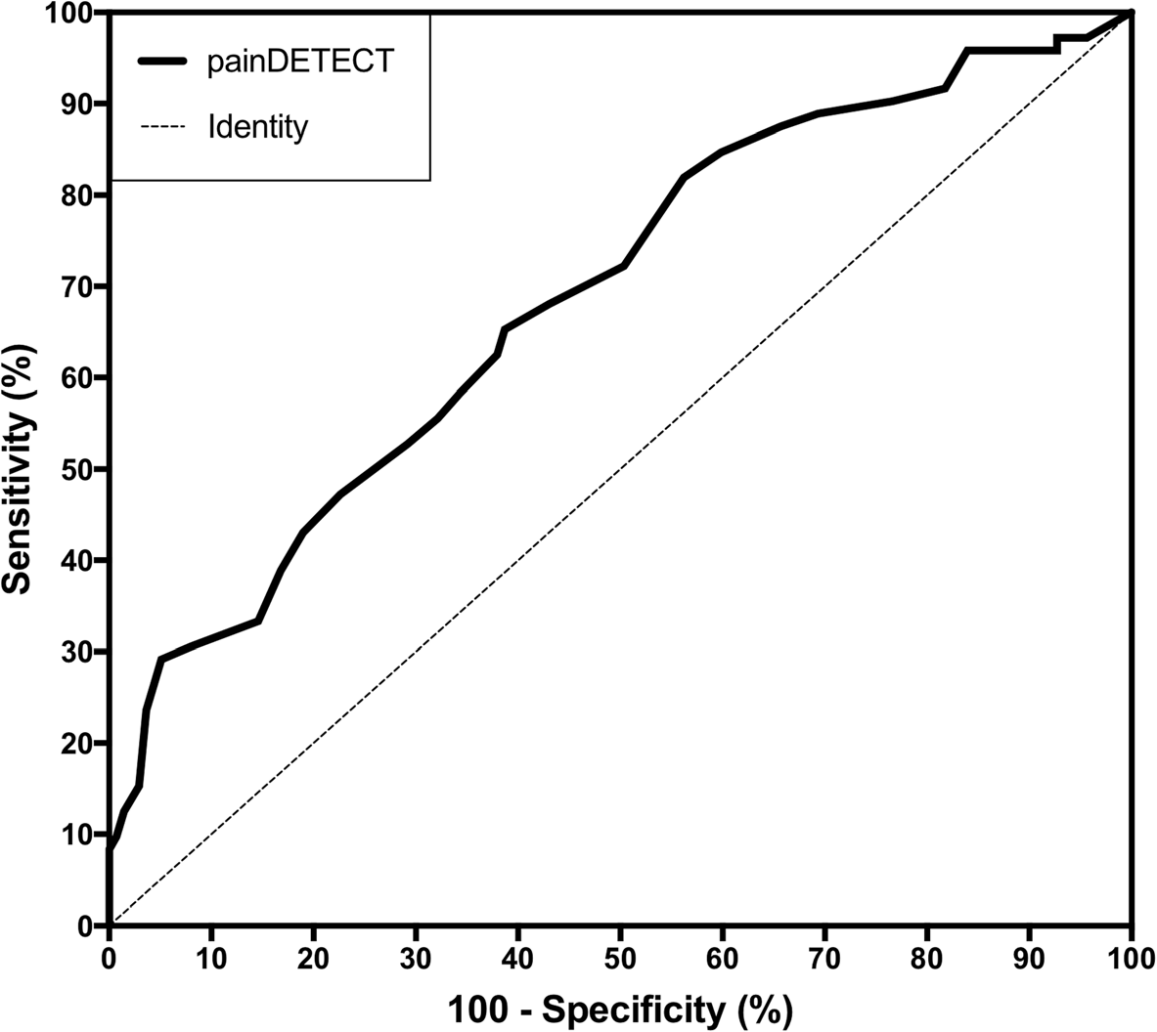
AUROC analysis of the PD-Q for detecting neuropathic pain components in the cohort. The AUC is compared to that of an identity line, with an area of 0.5. The difference in areas between the curve and the identity line was significant (*p* = 0.001)

ROC curve analysis was performed to determine the accuracy of the PD-Q in detection of neuropathic pain components. The AUC was calculated and compared to an identity line, with an area of 0.50, and sensitivities and specificities were derived for each cut-off of the PD-Q (Fig. 2). The AUC of the PD-Q was significantly higher than that of the identity line (AUC, 0.63; 95% CI, 0.58–0.70; *p* = 0.001). Our statistical model derived sensitivities and specificities corresponding to the PD-Q scores, and predictive values were calculated from these, given the prevalence of neuropathic or non-neuropathic pain within the patient cohort. At a cut-off of 11.5, given a prevalence of 54.6% patients without neuropathic pain components in the cohort, the PD-Q had a sensitivity of 59.6%, specificity of 56.9%, PPV of 62.4% and NPV of 53.5%. At a cut-off of 19.5, given a prevalence of 45.4% patients with some neuropathic pain components in the cohort, the PD-Q had a sensitivity of 28.9%, specificity of 83.2%, PPV of 58.9% and NPV of 58.5%.

**Fig. 3:**
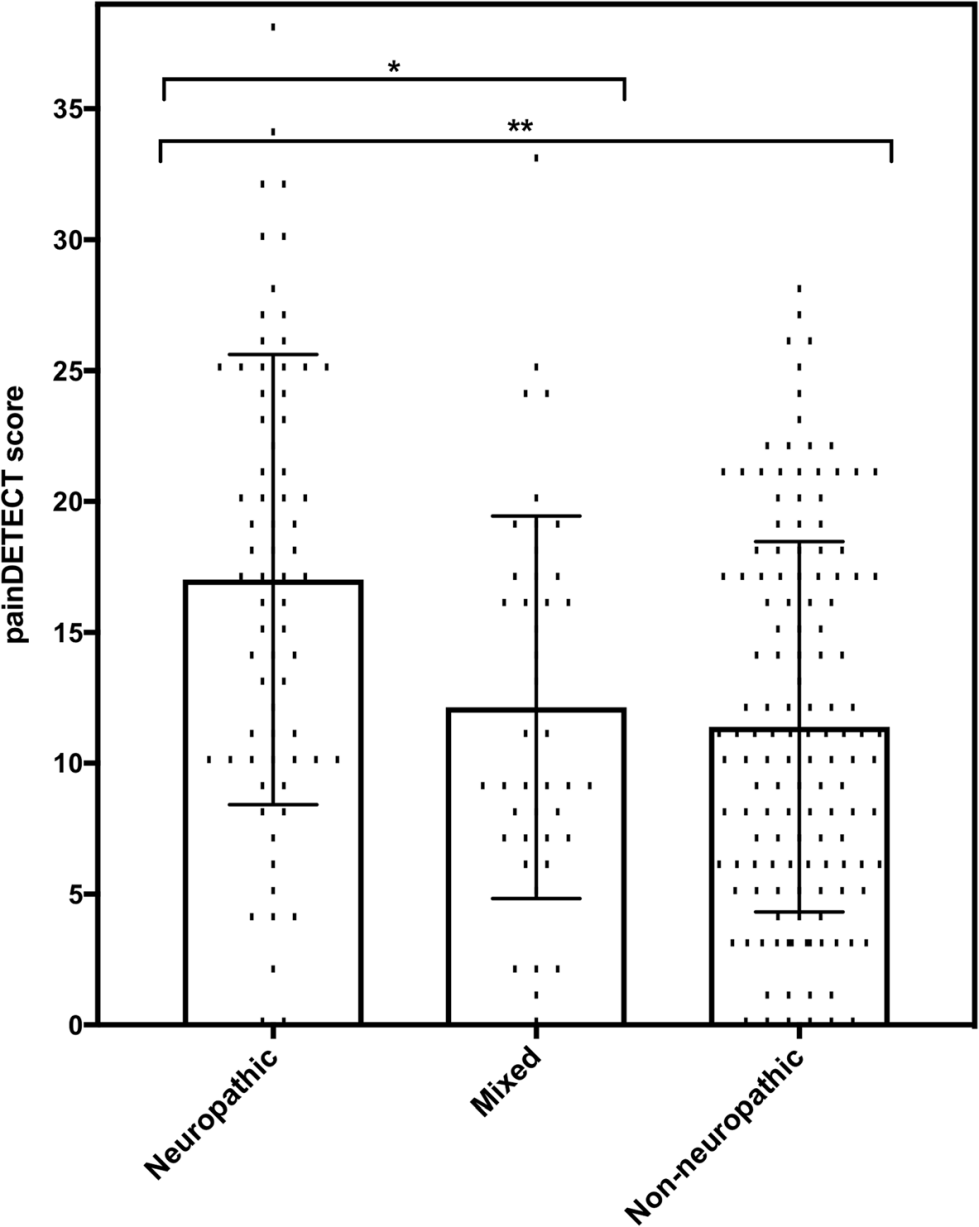
Scatterplot representing median PD-Q scores for each type of pain in the OFP cohort. Error bars indicate IQR. Brackets and asterisks represent statistically significant differences between median PD-Q scores. *: *p* < 0.05, **: *p* < 0.01

PD-Q scores were compared between the five most common OFP diagnoses within the cohort, using a Kruskal-Wallis test **(**Fig. 3**).** Overall, there was a significant difference (*p* < 0.001) between median PD-Q scores of patients with neuropathic pain (median, 17.0; IQR, 10.0–24.0), non-neuropathic pain (median, 11.0; IQR, 6.0–17.0) or mixed pain (median, 10.0; IQR, 7.0–17.0) aetiologies of OFP. Pairwise comparisons revealed statistically significant differences in median PD-Q score between neuropathic and non-neuropathic pain (*p* < 0.001) and between neuropathic and mixed pain (*p =* 0.008), but not between non-neuropathic and mixed pain (*p* > 0.5). The median PD-Q scores for the 12 most common clinical diagnoses, containing five or more patients per group and accounting for 212/251 (84.5%) of the cohort, are shown in Table 3**.**

**Table 3:** painDETECT scores grouped by clinical diagnosis

Type of pain	Clinical diagnosis	*n*	Median painDETECT score	IQR
Neuropathic	Trigeminal neuralgia	27	17.0	11.0–21.0
	Trigeminal neuropathic pain	21	17.0	10.0–26.5
	Burning mouth syndrome	6	9.5	8.3–16.8
	Trigeminal neuralgia with concomitant pain	5	17.0	8.0–18.0
	Short unilateral neuralgiform headache attacks with autonomic features	5	27.0	25.0–31.0
Non-neuropathic	Temporomandibular disorder	88	10.5	5.0–17.0
	Pericoronitis	8	11.0	10.3–14.0
	Psychosomatic	8	17.5	6.8–21.0
	Migraine	5	12.0	3.0–21.0
Mixed	Atypical odontalgia	20	8.0	5.3–14.0
	Chronic idiopathic facial pain	13	16.0	8.5–18.0
	Chronic post-dental treatment	6	12.5	5.3–24.0

### Patient factors associated with PD-Q score in orofacial pain

We performed a multivariate linear regression to determine whether any of the patient characteristics including: age, gender, secondary diagnosis or presence of anxiety or depression had an influence on the PD-Q score independently. There was a significant correlation between the PD-Q score and a secondary diagnosis (*r =* − 0.20; *p =* 0.001) and also anxiety or depression (*r =* − 0.15; *p =* 0.009). However, when adjusted in the regression model, only a secondary diagnosis contributed significantly to the PD-Q score (β = − 0.18; *p =* 0.006) when adjusted for patient age, gender and presence of anxiety or depression. Anxiety or depression, when adjusted for other patient characteristics, did not reach significance (β = − 0.12; *p =* 0.055).

## Discussion

This prospective study tested the accuracy of the PD-Q in identifying neuropathic pain components in a hospital-based cohort, with a broad range of orofacial pain diagnoses. At the PD-Q score above which neuropathic components are likely, the PD-Q had a low sensitivity and high specificity. Conversely, at the lower PD-Q cut-off, the PD-Q has a modest sensitivity and specificity. The PPVs and NPVs were modest at both cut-offs, indicating a reasonable likelihood that patients with a score above 19 would have a neuropathic pain component, and that patients with a score below 12 would not. PD-Q scores were significantly different between clear neuropathic OFP diagnoses, such as TN or TNP, compared to non-neuropathic diagnoses, such as TMD, whereas mixed diagnoses such as CIFP were more ambiguous. Together, these data suggest that the PD-Q identifies neuropathic components when clear-cut, but unsurprisingly, performs less well in patients with a complex, mixed diagnosis, particularly when multiple diagnoses are present.

Previous studies have examined the utility of the PD-Q for OFP diagnoses in tertiary centres, and suggest the PD-Q may not be an appropriate tool in this context. Elias and colleagues found that only 34% of patients with post-traumatic trigeminal nerve injury obtained a PD-Q score of at least 19 [13]. Heo and colleagues applied the PD-Q to patients with BMS, and found a low sensitivity (16.7%) and high specificity (97.4%) at a cut-off of 19 [14]. These studies, with smaller sample sizes, include patients with predominantly neuropathic pain, and their findings may reflect the low sensitivity of the PD-Q at the higher cut-off value in the present study. In contrast, our ROC analysis suggested that the PD-Q has potential for recognition of neuropathic pain in this hospital-based cohort, likely because our patient population is more heterogeneous and representative of secondary care.

Unlike other questionnaires for neuropathic pain, the PD-Q does not involve clinical examination. Such examination, including changes in sensory perception, is critical for making a diagnosis of a neuropathic pain [12]. The PD-Q was originally designed to identify neuropathic components in lower back pain [10]. Though response rates in this study reflect the ease of completing the questionnaire, the design of the PD-Q makes it difficult for patients to highlight and draw areas where pain predominates and radiates to, particularly considering as the size of the head is very small in the figure within the PD-Q. Questions in the PD-Q referring to possible pain triggers do not account for specific face pain triggers such as washing the face, showering compared to bathing, or the cold wind; all of which are clues towards orofacial pain of a neuropathic aetiology, such as classic TN [21].

Despite the strengths of this study, including its prospective nature, blinding of the clinician to the questionnaire results and the confirmation of the reference standard of clinical diagnosis by an independent clinician, our data should be interpreted with caution. Firstly, the study was conducted in a secondary care centre, receiving population of orofacial pain patients not representative of primary care or non-specialist settings. Data previously published from this centre indicated that up to 46% of the patients seen have a diagnosis of TMD [5], whereas its estimated prevalence in the general population is between 2 and 6% in developed countries [22]. Given this prevalence, the rarity of conditions such as TMD and TN would make a prospective study in primary care extremely challenging. Another difficulty in translating these results to primary care is the possibility of changes to the way the questionnaire is filled out in different settings. In different settings, patients may rate their pain variably, dependent on their expectations and desired outcomes of their consultation. Other factors differ between centres, such as the person administering the measure, be they clinician, family member or study investigator. Moreover, only a small number of patients with acute dental pain were recruited, which contrasts with primary dental care in which acute dental pain is predominant. However, the inclusion of these patients demonstrated that acute dental pain is not classified as neuropathic, and demonstrate that patients who score highly in primary dental care should be referred to a specialist centre for appropriate management of neuropathic pain. A second limitation is the difficulty in accommodating for the large proportion of patients in each group with a secondary diagnosis. This is representative of the complexity of orofacial pain presentations, and considerably influences the ability of the PD-Q to accurately identify OFP aetiology, independent of other patient factors, but likely influences non-adjusted analyses. To accommodate for this, study clinicians made a primary diagnosis based on patient history and examination, to determine the predominant type of pain. The study is further limited by characteristics not recorded, such as the pain intensity or educational level of patients, both of which could influence PD-Q scores. Though patients were recruited consecutively over individual study periods, the nature of the study, namely the periods of time during which patients were not recruited due to absence of postgraduate students, may have introduced selection bias to the sample. Re-test validity was not included in this study. Finally, the clinician confirming the diagnosis, though independent, was not blinded to diagnosis made at first consultation, and may be biased by this information or by treatment response. The time between diagnosis made at first consultation and independent confirmation was not recorded.

The accuracy of the PD-Q is only one of the considerations when determining a screening tool for OFP. The PD-Q appears a valid tool, in its effectiveness in distinguishing neuropathic from non-neuropathic pain in other contexts [11], the continuous score of the PD-Q reflecting the spectrum of neuropathic pain presentations [3] and its availability and validation in different languages. What has not been compared is the ultimate treatment and outcome of patients and how these relate to the initial PD-Q scores, which could be considered its criterion validity. Moreover, the reliability of the PD-Q in patients with OFP needs to be ascertained prior to its implementation in practice. Preliminary data at our centre indicates a strong concordance in PD-Q score before and after consultation with a facial pain clinician, but larger sample sizes are needed to validate this. The utility and performance of the PD-Q could also be compared to other screening tools for neuropathic pain [12], and more specific screening tools for OFP diagnoses, such as those available for TMD and TN [23, 24]. Finally, the differences between settings prompt a revalidation of the PD-Q in primary care [25], given the considerably lower prevalence of specific OFP diagnoses in general clinical and dental practice.

## Conclusions

Patient-completed screening tools, such the PD-Q, have promise in both primary care and hospital practice, given their ease of use, high completion rate and the potential to aid the triaging of patients with OFP prior to consultation. Such tools may help to identify patients in primary care who need a specialist referral, those in dentistry who have a non-odontogenic origin of their pain or may help to inform clinicians as to the aetiology of pain to make earlier decisions about management and therapy. However, the PD-Q performed modestly in our centre given the complexity of presentation and as many patients have more than one co-existing diagnosis. Prior to clinical and further research applications, the PD-Q must be adapted and revalidated for orofacial pain patients, and separately in primary care, where orofacial pain is considerably less common. Ultimately, either patient-completed screening tools should only be implemented within settings they were designed, or pre-existing general screening tools needs to be optimised in different settings to reflect the variety of clinical situations for which such tools may be applicable.

## Additional file

Additional file 1: **Table S1.** Secondary clinical diagnoses within the OFP cohort. (DOCX 16 kb)

## Abbreviations

AUC: area under the curve
BMS: burning mouth syndrome
CI: confidence interval
CIFP: chronic idiopathic facial pain
IQR: interquartile range.
OFP: orofacial pain
PD-Q: painDETECT screening questionnaire
ROC: receiver operating characteristics
SD: standard deviation
TMD: temporomandibular disorder
TN: trigeminal neuralgia
