# Supplementary material for: Accuracy of the painDETECT screening questionnaire for detection of neuropathic components in hospital-based patients with orofacial pain: a prospective cohort study
Source: J Headache Pain. 2018 Nov 6;19(1):103. doi: 10.1186/s10194-018-0932-5 (PMC6755558; doi:10.1186/s10194-018-0932-5)
Supplement: Supplementary file 1 — Table S1. Secondary clinical diagnoses within the OFP cohort. (DOCX 16 kb) [file 10194_2018_932_MOESM1_ESM.docx]

**SUPPLEMENTARY MATERIAL**

**Table S1. Secondary clinical diagnoses within the OFP cohort**

| **Clinical diagnosis** | **Frequency** |
| --- | --- |
| Atypical odontalgia | 1 |
| Burning mouth syndrome | 1 |
| Cardiac problems | 1 |
| Chronic idiopathic facial pain | 3 |
| Chronic tension headache | 7 |
| Dental pain | 4 |
| Ear pain / tinnitus | 2 |
| Ehlers Danlos syndrome | 1 |
| Fibromyalgia | 7 |
| Gastrointestinal complaints | 1 |
| Hypervigilance | 1 |
| Migraine | 15 |
| Medication overuse headache | 3 |
| Myalgia | 1 |
| Nasal problems | 1 |
| Neck pain | 1 |
| Other non-odontogenic pain | 3 |
| Psychosomatic | 3 |
| Sleep apnoea | 1 |
| SUNA | 1 |
| Temporal arteritis | 1 |
| Temporomandibular disorder | 6 |
| Tourette’s syndrome | 1 |
| Trigeminal neuralgia | 1 |
| Trigeminal neuropathic pain | 7 |
